# Age-specific risk factors for the prediction of obesity using a machine learning approach

**DOI:** 10.3389/fpubh.2022.998782

**Published:** 2023-01-17

**Authors:** Junhwi Jeon, Sunmi Lee, Chunyoung Oh

**Affiliations:** ^1^Department of Applied Mathematics, Kyung Hee University, Yongin, South Korea; ^2^Department of Mathematics Education, Chonnam National University, Gwangju, South Korea

**Keywords:** obesity prediction, machine learning, age-specific, gender-specific, risk factors, KNHANES

## Abstract

Machine Learning is a powerful tool to discover hidden information and relationships in various data-driven research fields. Obesity is an extremely complex topic, involving biological, physiological, psychological, and environmental factors. One successful approach to the topic is machine learning frameworks, which can reveal complex and essential risk factors of obesity. Over the last two decades, the obese population (BMI of above 23) in Korea has grown. The purpose of this study is to identify risk factors that predict obesity using machine learning classifiers and identify the algorithm with the best accuracy among classifiers used for obesity prediction. This work will allow people to assess obesity risk from blood tests and blood pressure data based on the KNHANES, which used data constructed by the annual survey. Our data include a total of 21,100 participants (male 10,000 and female 11,100). We assess obesity prediction by utilizing six machine learning algorithms. We explore age- and gender-specific risk factors of obesity for adults (19–79 years old). Our results highlight the four most significant features in all age-gender groups for predicting obesity: triglycerides, ALT (SGPT), glycated hemoglobin, and uric acid. Our findings show that the risk factors for obesity are sensitive to age and gender under different machine learning algorithms. Performance is highest for the 19–39 age group of both genders, with over 70% accuracy and AUC, while the 60–79 age group shows around 65% accuracy and AUC. For the 40–59 age groups, the proposed algorithm achieved over 70% in AUC, but for the female participants, it achieved lower than 70% accuracy. For all classifiers and age groups, there is no big difference in the accuracy ratio when the number of features is more than six; however, the accuracy ratio decreased in the female 19–39 age group.

## 1. Introduction

The prevalence of obesity has become one of the most prominent issues in global public health. The causes of obesity fall into several categories, including physiology, individual psychology, food production, food consumption, physiology, individual physical activity, genetic and cultural influence, and physical activity environment (1, 2). With the number of obese people doubling in two decades (from 1.3 million people obese globally in 1980 to 2.6 million in 2008), unhealthy habits, unhealthy diets, high intake of saturated fat, discretionary foods, and physical inactivity are the major factors causing “obesity and overweight” (3). The medical problems caused by obesity increase the risk of other diseases and health problems, such as heart disease, diabetes, high blood pressure, and certain cancers (4, 5). Moreover, obesity can diminish the overall quality of a person's life (6).

Obesity is responsible for a large fraction of costs to both the healthcare system and society at large. Diabetes, cancer, cerebrovascular disease, hypertensive disease, and arthrosis are diseases related to obesity, which resulted in socioeconomic costs of about KRW 1.36 trillion in long-term socioeconomic costs associated with adolescent obesity (7). As of 2016, social costs, such as medical and nursing expenses due to obesity, were estimated at 11.5 trillion (KRW) per year by Korea (8). The review for Europe which encompassed both direct and indirect costs estimated obesity-related costs to range from 0.09 to 0.61% of total annual gross domestic income in Western European countries (9, 10). Moreover, social and economic factors are linked to obesity. If the costs of illness attributable to obesity could be minimized, monetary resources within national healthcare systems and economies could be reallocated toward other uses.

**Figure 1:**
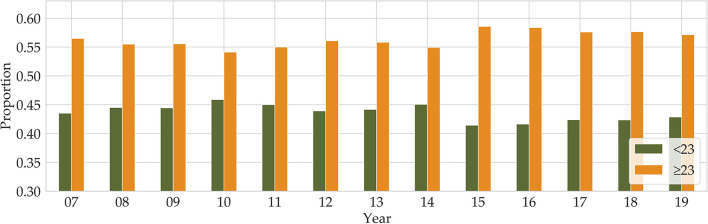
Annual proportions of BMI below and above 23 are shown from 2007 to 2019 (Orange for BMI above 23 and green for BMI below 23).

Until the 1970s, obesity was defined against an ‘ideal body weight', derived from actuarial tables compiled by the life insurance industry. The Body Mass Index (BMI) in adults is defined as the ratio of body mass in kilograms to the square of the individual's height in meters. In the 1980s, the ideal body weight approach was replaced by BMI (*kg*/*m*^2^) (11), and the commonly used cutoffs for normal weight (BMI:18.5~22.9), overweight (BMI: 23~24.9), and obesity (25 ≤ BMI), for both men and women, were adopted to define obesity in adults according to the Asia-Pacific guidelines (12, 13). Even though there can be confusion and misinformation associated with BMI, BMI is the most commonly used measure of adiposity in epidemiological research (14). We used BMI as our obesity criterion. The Korea National Health and Nutrition Examination Survey (KNHANES) has been conducted since 1998, and the BMI factor is included from 2007. The KNHANES is one of the principal sources for investigating obesity in the population, with many variables and vast amounts of data related to obesity-influenced diseases. According to the BMI data of the KNHANES, in 2007, 23% of adults were overweight and 32% were obese, while in 2019, 23% were overweight and 34% were obese. Over the 12 years, obesity increased by 2%, while there was no increase in the proportion of overweight adults^1^ (see Figure 1). Overall, there are more people who are overweight or obese than those with a BMI below 23.

Recently, the use of machine learning models for disease classification has been developing rapidly, both because of the significant amount of data that is being generated by healthcare devices and systems and the magnitude of computational resources available for data calculation and processing (15–17). Obesity researchers and healthcare professionals have access to a wealth of data. Importantly, this immense volume of data is utilized to train models and facilitated the use of expert systems, machine learning techniques, and classification techniques for finding trends and patterns in the evaluation and classification of several diseases (18). Machine learning techniques applied to large survey datasets may provide a meaningful data-driven approach to categorizing patients for population health management, which is a critical part of combating obesity. Machine learning algorithms can be applied to assess the factors leading up to the prevalence of obesity. Our approach to estimating obesity was to use machine learning on the South Korean KNHANES datasets to estimate obesity.

As obesity increases, so does the risk for a variety of diseases. Identifying suspected clinical findings among the top-ranked factors is important to preventing obesity. In this article, we aimed to identify the metabolic factors affecting BMI or obesity in the KNHANES datasets, and to predict obesity using various machine learning algorithms. First, we carried out a correlation among the risk factors for obesity. Second, we split the KNHANES dataset into six groups; three age groups each split into two genders. Third, we employed six machine learning algorithms: support vector machine (SVM), logistic regression (LR), random forest (RF), multi-layer perceptron (MLP), light gradient boosting (LGBM), and extreme gradient boosting (XGB). In addition, we measured the performance of machine learning classification algorithms in terms of performance metrics such as specificity, accuracy, AUC, precision, recall, and F1-score. Furthermore, we identified the eight most important age- and gender-specific risk factors through feature importance methods (random forest and sharp value). We also determined the impact of this top eight ranked risk factors on the performance, accuracy, and AUC of the machine learning algorithms.

## 2. Literature review

Since the causes of obesity are very diverse, numerous studies have tried to predict obesity. Different machine learning methods have been used to predict obesity using a wide range of input factors.

We examined the results of previous studies that predicted obesity by applying various classifiers of machine learning to various factors involved in adult obesity and compare and differentiate them with our study results (see Table 1). Reviewing many research articles, there is no research that applied machine learning algorithms to predict obesity from mainly metabolic factors (Table 1).

**Table 1:** Machine learning models and the risk factors related to adult obesity.

**References**	**Type of model**	**Risk factors**	**Best classifier evaluation**
This study	MLP, RF, LR, LGBM, XGB, SVM	Eight items of (ALT, glu, TG, sbp, dbp, Ast, HbA1c, chol, Bplt, WBC, RBC, crea, PLS, and UA) in KNHANES	19–39 age(male, female) MLP: AUC = (77, 78)% RF:accuracy = (72, 76)%
Cheng et al. (19)	LR, Naïve Bayes, J48, Radial Basis Function, J48, Random subspace, Local KNN, MLP, Decision table Random tree	physical activity in NHANES	random subspace: accuracy = 70.01%, AUC = 63.3%
Thamrin et al. (20)	LR, Naïve Bayes, CART	location, marital status, age group, education, work category, sugary foods, sweet drinks, instant foods, energy drinks, salty foods, fatty/oily foods, grilled foods, preserved foods, smoking, seasoning powders, soft/carbonated drinks, alcoholic drinks, mental-emotional disorders, diagnosed hypertension, physical activity, fruit and vegetables consumptions, RISKESDAS	LR: accuracy = 72.22%, AUC = 79.79%
Ferdowsy et al. (21)	KNN, RF, LR, GB, MLP, SVM, DT, ADAB, Naïve Bayes, GBM	Daily activities, Food routines, Height, Weight	LR: accuracy = 97.09%
Jindal et al. (5)	Ensemble utilized RF	Age, Height, Weight, BMI	ensemble: RF accuracy = 89.68%
Montañez et al. (22)	GBM, GLMNET, RF, KNN, SVMRadial, CART, NNET	Age, Gender, Genetic variants or single Nucleotide Polymorphisms (13-SNPs)	SVM: AUC = 90.5%.

Table 1 displays the comparison between our work and other works. Cheng et al. (19) classified factors using data from the NHANES. They predicted whether an individual was overweight or obese based on physical activity levels. They used 11 algorithms for the estimates. They obtained the best performance from the random subspace classifier, which had the highest overall accuracy of 70.01 and 63.3% area under the ROC curve (AUC). Thamrin et al. (20) used three models for 22 features in predicting obesity status in adults. The logistic regression classifier had the highest accuracy of 72.22% and produced the highest result with 79.79% area under the ROC curve (AUC), with a 10-fold CV for performance evaluation. Ferdowsy et al. (21) applied nine machine learning algorithms to four features. The logistic regression algorithm achieved the highest accuracy of 97.09%. Jindal et al. (5) performed ensemble machine learning approaches for obesity prediction based on the four key determinants. The ensemble model utilized a random forest, a generalized linear model, and partial least squares, with a prediction accuracy of 89.68%. To predict the future risk of developing complex diseases such as obesity, from ones BMI status and SNP profile, Abdulaimma et al. (22) introduced a genetic profile predictive study using machine learning algorithms and used the publicly available participants' profiles, genetic variants, and Single Nucleotide Polymorphisms (SNPs). Seven machine learning algorithms for the prediction of obesity were used on the 13 SNPs, and the support vector machine generated the highest area under the curve value of 90.5%.

**Figure 2:**
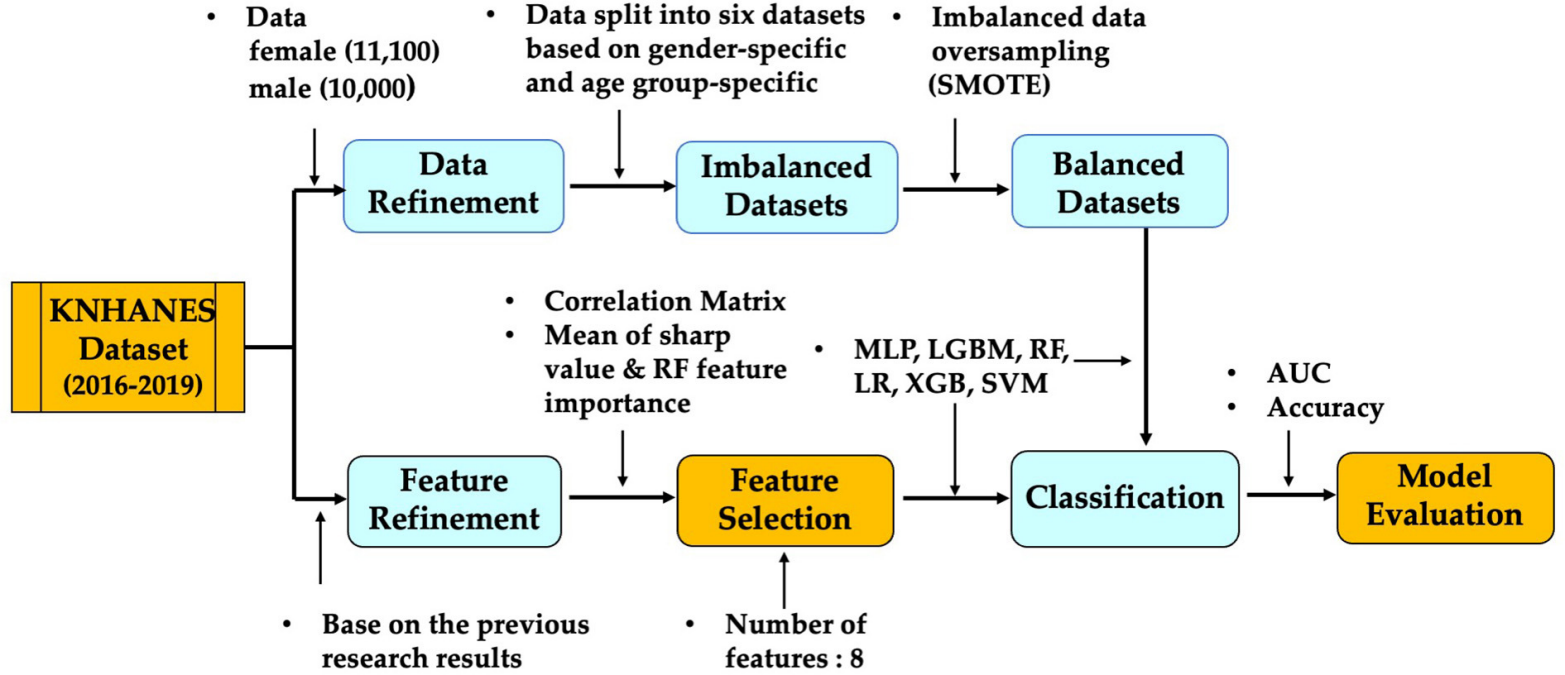
Schematic diagram for identifying and predicting obesity risk factors.

According to the works of the review literature on obesity, most studies on obesity prediction used features focused on certain aspects of the participants' lifestyles or behavior. Unlike the variables selected in previous studies, we tried to predict obesity by selecting variables that are involved in the metabolic cause of obesity. Our research utilized participants' metabolic factors in the KNHANES and we compare them with our study results. Figure 2 presents a schematic diagram for identifying and predicting obesity risk factors using machine learning algorithms.

## 3. Descriptions of data and features

### 3.1. Data sources

Our datasets are prepared and published through the Korea National Health and Nutrition Examination Survey (Korea Division of Health and Nutrition Survey and Analysis, KDCA), to provide full access. The Korea National Health and Nutrition Examination Survey (KNHANES) is a national program that is designed to assess the health and nutritional status of adults and children in Korea. Since 1998, the KNHANES has collected data obtained by direct physical examination, clinical and laboratory tests, personal interviews, and related measurement procedures. The KNHANES was conducted on a triennial basis from 1998 to 2005. In 2007, the survey became a continuous, annual survey program conducted by the KDCA, and many new health measurements were added to the basic design to meet emerging data needs (23). In 2007, BMI was added, and uric acid has been included as a survey variable since 2016 (see text footnote 1, respectively).

Two stages of stratified clustering, consisting of primary sampling units and households, were applied to the data collected from the population and housing census in Korea. In general, blood and urine samples are collected from participants aged 10 years and above (24). The extent of examination differs, depending on the age of the participant, but targeted individuals start at the age of 1 year (see text footnote 1, respectively). These data are used to estimate the prevalence of chronic diseases in the total population or monitor trends in the prevalence and risk behaviors. Approximately 10, 000 people are sampled in total, in all 192 variables for the primary sample units per year (23). The data are composed of demographic variables, health questionnaires, medical examination, and a nutritional survey (see text footnote 1, respectively) (23). The data in the KNHANES consist of categorical data, numeric data, and text data, and we refined the numeric data into obesity risk factors. The dataset can be accessed at the Korea National Health and Nutrition Examination Survey (see text footnote 1, respectively).

The KNHANES is a national program that is designed to assess the health and nutritional status of adults and children in Korea. Obesity is defined as BMI above 25 according to the Asia-Pacific adult guidelines (12). In our data, we combined obese (BMI of >25) and overweight (BMI of >23 and BMI of <25) into our definition of obese. After removing missing values, the total number of participants was about 21,100 (male 10,000 and female 11,100). As can be seen from Figure 1, the proportion of overweight and obese people between 2016 and 2018 was 58%, and in 2019, the proportion was 57%. After we split the dataset into two parts by gender, we divided both the male and female participants into three age groups (19–39, 40–59, and 60–79), respectively. Therefore, our dataset consists of six groups composed according to gender and age group.

### 3.2. Oversampling

In machine learning applications, data preprocessing plays a significant role in achieving better performance and accurate results. Our data comprises of the KNHANES participants from 19 to 79 years of age between 2016 and 2019. We cleaned the data by excluding all records with incomplete or missing values for the variable/feature BMI, a core feature used to categorize obesity status. After we cleaned missing values, there were about 21,100 participants for the top selected features. After we split the dataset into two parts by gender, we divided both male and female participants into three age groups (19–39, 40–59, and 60–79), respectively.

As overweight and obese categories are both at risk, instances belonging to these two classes have been combined and labeled as ‘BMI'. Although this slightly reduces the imbalance, most of the algorithms are only able to classify the majority class with a high degree of accuracy. One of the factors that deteriorates the accuracy of the test dataset in AI classification modeling is class imbalance. If the model is trained using data where the proportion of each class is significantly different, the predictive model tends to be biased toward a specific class or has difficulties evaluating properly, meaning imbalance acts as a factor of performance degradation. Conducting data analysis in a highly imbalanced dataset is not trivial, and often leads to low sensitivity results being obtained (18). Our dataset consists of six groups that are composed of gender and age groups. Supplementary Table S1 illustrates that most of the participants belong to the obesity category, which makes the datasets imbalanced. The Synthetic Minority Over-Sampling Technique (SMOTE) is one of the representative oversampling techniques. It is a method to create a new sample and add it to the data by using the KNN (*k*≥2) algorithm from samples of an underrepresented class (25). In this study, the SMOTE technique with oversampling was used, which resulted in two new datasets with 50% obese and 50% non-obese entries. Each dataset used the training part to train the model (75%) and the testing part to test the model (25%).

## 4. Results

### 4.1. Feature selection

**Figure 3:**
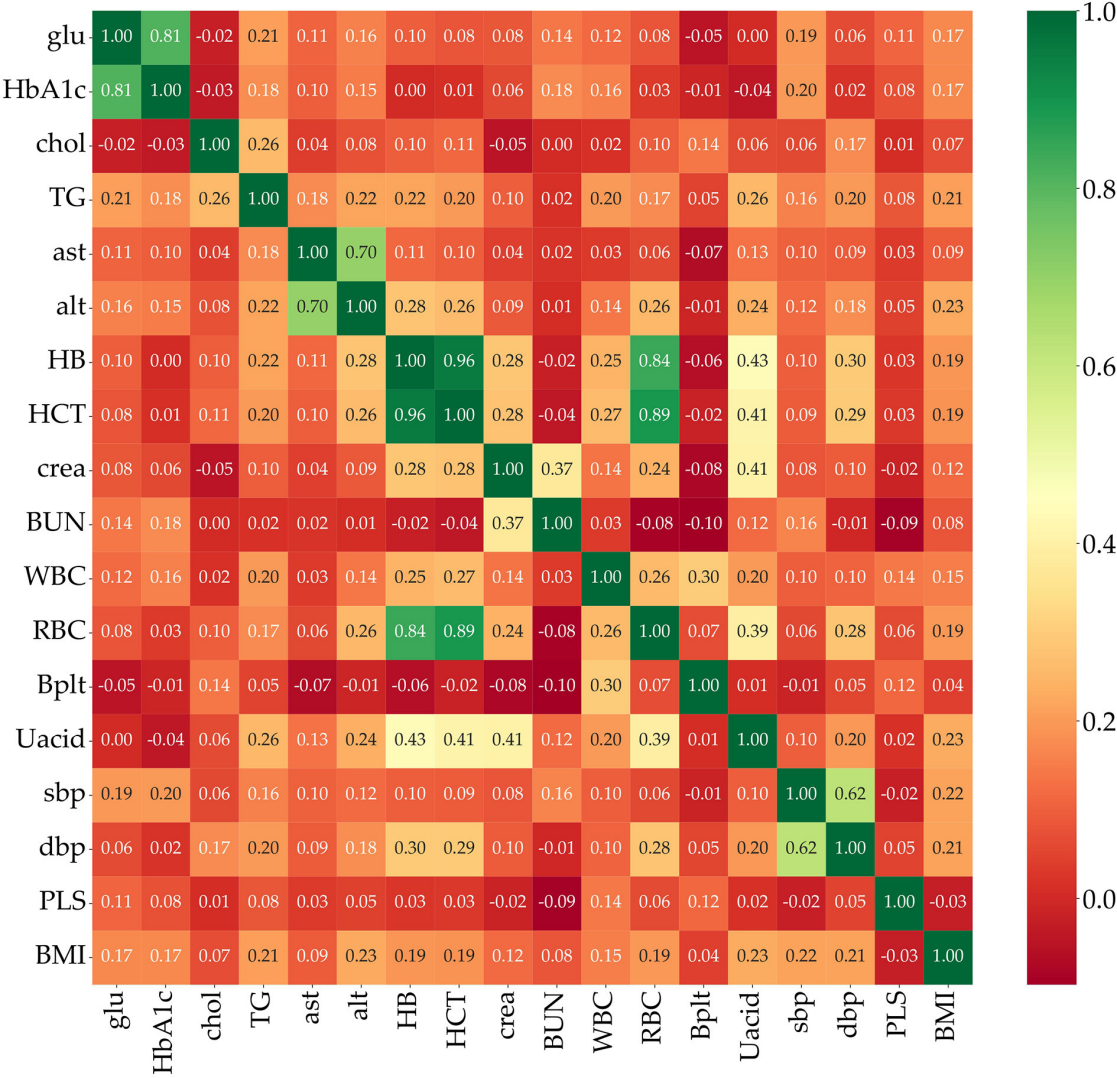
Correlation between BMI and 17 selected metabolic features given in the KNHANES.

**Figure 4:**
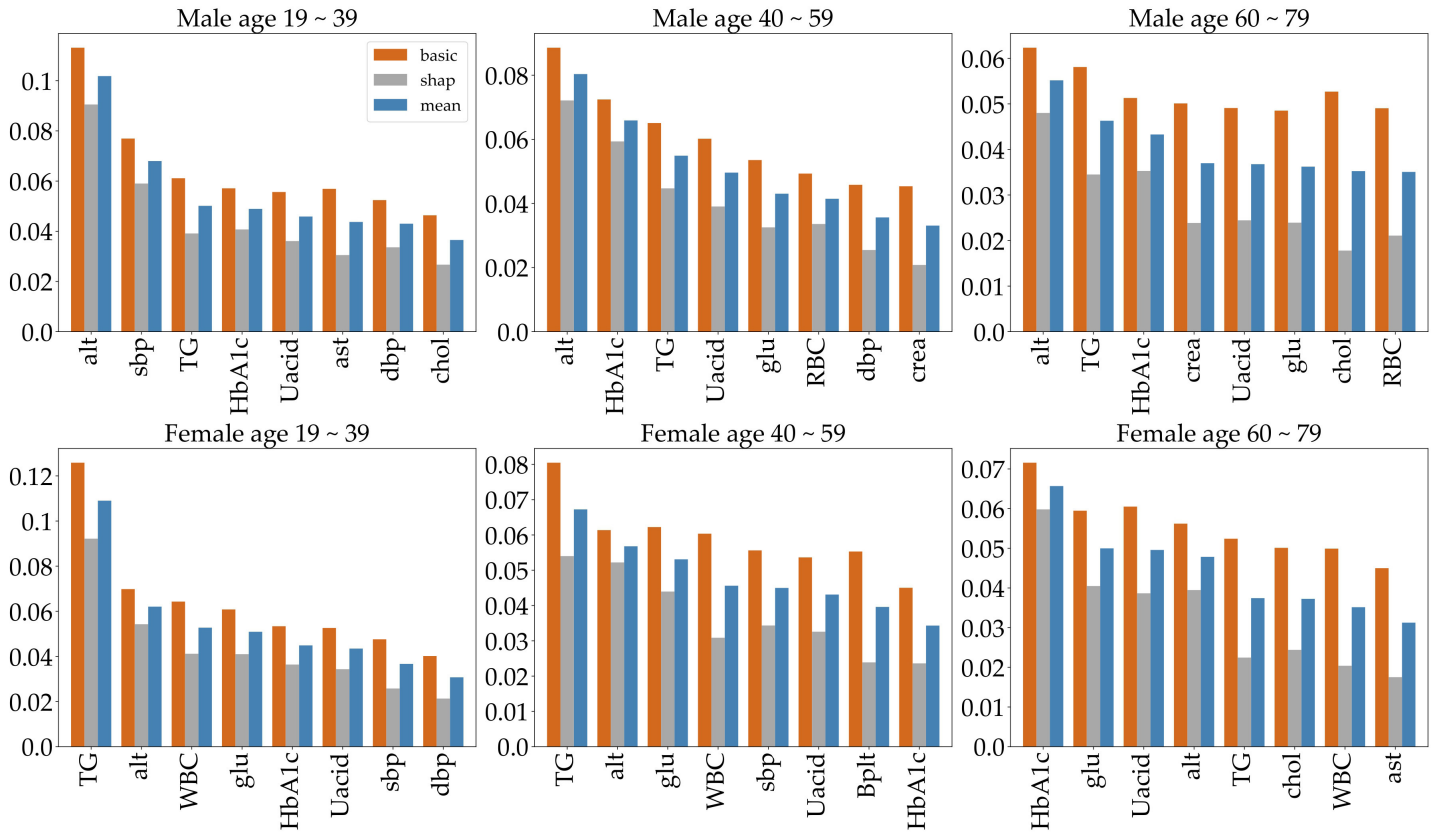
The top eight important features, selected by age group and gender. To indicate feature importance, the orange bar represents the random forest value, and the gray bar represents the average absolute sharp value. The blue bar shows the mean of the two values.

Feature selection is used to identify the set of features that are most important to the performance of the classifiers. Obesity is caused by many factors, and a great many studies have identified various factors that cause obesity. However, obesity prediction using all factors in the KNHANES with models did not show reliable performance. We chose risk factors according to the following procedure. There are 192 variables in the KNHANES in total. Among these variables, 19 were blood test variables and 3 were blood pressure variables. These variables are quantitative variables. First, we found metabolic variables and pulse pressure that affect obesity or BMI increases based on previous studies. Table 2 shows the 15 influential factors included in the KNHANES. From Ko and Oh (42), one blood test variable (BUN) was added. Since being overweight or obese is positively associated with pulse pressure, independently of age and gender (43), we include one more factor of pulse pressure in our features. Therefore, we narrowed down to just 17 factors out of the KNHANES contributing to BMI or obesity. Next, we computed the correlation coefficients between the selected 17 independent variables and the dependent variable BMI. Figure 3 depicts the correlation among the 17 selected features and illustrates the correlation coefficient between the selected variables and the target feature, BMI. The final selected features have a significant relationship for the correlation coefficient between the 17 variables and BMI. Finally, we carried out age-specific feature selection for each male and female participants. We selected the top eight ranked factors out of 17, from the ranking of the mean of the random forest values and SHAP values (see Figure 4). We conducted feature importance analysis through a tree model, such as a random forest. The disadvantage is that Gini importance for calculating feature importance in random forests can be biased due to the number of predictor categories and the size of the measurement (44). We additionally used SHAP feature importance. Shapley Additive explanation (SHAP) values have been proposed for SHAP values as a unified measure of feature importance (45). SHAP feature importance defines feature importance as a mean absolute SHAP value. Each time a feature is added, the contribution is calculated and averaged. When used for tree-based models, SHAP has the great advantage of being able to calculate Shapley values relatively quickly (46). The selected features are made up of slightly different features according to age group and gender. Therefore, we utilized SHAP to identify the principal features in model prediction. we used the mean of the random forest value and mean absolute Shap value, and selected eight factors for each of the six age-specific groups. The selected features were slightly different for each of the six population groups. A total of 13 features, ALT (SGPT), glucose (glu), triglyceride (TG), white blood cell (WBC), glycated hemoglobin (HbA1c), creatinine (cre), systolic (diastolic) blood pressure (sbp/sdp), AST (SGOT), cholesterol (chol), platelet (Bplt), RBC, and uric acid (UA), are given in Figure 4. Variables in Figure 4 were selected in the order of the highest correlation coefficient with BMI in Figure 3.

**Table 2:** Metabolic factors causing BMI or obesity based on previous studies.

**Feature name**	**References**	**Feature name**	**References**
Serum creatinine (cre)	(26)	Hemoglobin (Hb)	(27)
Red blood cell (RBC)	(28)	ALT(SGPT)	(29), (30)
Fasting serum glucose (glu)	(31)	Uric acid (UA)	(32), (33)
Ast (SGOT)	(34)	Cholestol (chol)	(35)
Systolic (diastolic) blood pressure (sdp)/(dbp)	(4)	Triglyceride (TG)	(36), (37)
Glycated hemoglobin (HbA1c)	(38)	Platelet (Bplt)	(39), (40)
Hematocirit (Hct)	(28)	White blood cell (WBC)	(41)

### 4.2. Machine learning algorithms

The feature vectors selected in the above-mentioned process are fed to six classifiers for training, and then testing. These classifiers are random forest (RF), support vector machine (SVM), logistic regression (LR), multi-layer perceptron (MLP), light gradient boosting (LGBM), and extreme gradient boosting (XGB). We distinguish the classifier with the best accuracy and AUC through the performance of these classifiers. Each of these six algorithms has certain parameters, with values that vary across all the algorithms. These parameter values are used to train the model, and they are discussed in Table 3.

**Table 3:** Detailed specifications for the six algorithms.

**Algorithms**	**Specification of algorithms**
MLP	Alpha = [0.001, 0.001, 0.0001, 0.01, 0.01, 0.001]
	Activation : relu
	Hidden layer size [100, 100]
LGBM	Number of leaves = [70, 100, 100, 70, 3, 100]
	Maximum depth = [30, 30, 30, 30, 2, 30]
RFC	Maximum depth = [30, 30, 30, 30, 5, 30]
	num. estimators = [100, 100, 100, 70, 100, 100]
SVM	C = [50, 30, 30, 10, 50, 50]
	Gamma = [50, 50, 50, 50, 1, 50]
	Kernel: radial basis function
LR	Maximum num. iterations = 1,000
	Penalty = l2
XGB	Maximum depth =[30, 50, 30, 30, 2, 50]
	gamma = [0.2, 0.1, 0.1, 0.2, 0, 0]
	num. estimators = [40, 70, 40, 100, 40, 20, 50]

The order of [,] is according to male age and female age.

The classification problem is designed for binary output because our data will only consider a model for binary classification based on BMI. A multi-layer perceptron is made up of a large number of neurons. In general, it consists of three layers: an input layer, a hidden layer, and an output layer, with an activation function between each layer to give non-linearity. In supervised learning, since inputs and outputs are given, only the weights are updated. MLP utilizes backpropagation for training, which is a supervised learning technique (47). A support vector machine (SVM) is mainly used for classification and regression analysis and is a model that defines decision boundaries for classification. The SVM builds a maximum margin separator, which is used to make decision boundaries with the largest possible distance (48).

Logistic regression (LR) predicts the probability that data will fall into a certain category as a value between 0 and 1, and classifies it as belonging to a more likely category based on that probability. Logistic regression can be viewed as a classification technique because the dependent variable is intended for categorical data. In particular, LR is mainly used when the dependent variable is a binomial problem (49). Random forest (RF) is a kind of ensemble learning method that integrates predictions of multiple base models and outputs classification results from multiple decision trees constructed during the training process. So, random forest chooses a voting method that collects classification results from a number of decision trees configured through training to obtain a conclusion. A random forest can quickly build a model even when the size of the data is huge, and the decision tree, which is the base model, has the advantage that it does not require a premise for data distribution (50).

GBM is a generalized method of boosting to any differentiable loss function proposed by Friedman (51, 52). GBM mitigates inaccuracies, a major drawback of decision trees, by averaging many small trees (51). Light Gradient Boosting (LGBM) is a relatively new model and a gradient learning framework based on a decision tree and the idea of boosting (53). It uses histogram-based algorithms to speed up the training process. GBM adds a maximum depth limit to the top of the leaf to prevent overfitting while ensuring high efficiency. The Extreme Gradient Boosting (XGB) is derived from the Gradient Tree Boosting algorithm. XGB uses shrinkage and subsampling to prevent overfitting (54).

For each of the six datasets, we measured the performance of each classifier using the confusion matrix. One can check that the confusion matrix for a 2-class classification is a 2 × 2 matrix. Its entries are the number of true positives (TPs), false negatives (FNs), false positives (FPs), and true negatives (TNs) for a 2-class problem (21). True positives mean the number of positive examples that the model correctly classified as positive, true negatives mean the number of negative examples that the model correctly classified as negative, false positives mean the number of negative examples that the model incorrectly classified as positive, and false negatives mean the number of positive examples that the model incorrectly classified as negative. Table 4 defines sensitivity, specificity, and accuracy. F1-score is the measurement of the harmonic mean of recall and precision. The TP, TN, FP, and FN amounts are computed for each dataset using Table 4. We implement a binary classification of obese or not obese based on BMI. AUC or AUROC is area under ROC curve. The value of AUC characterizes the model performance. Higher the AUC value, higher the performance of the model. In our study, when classified as obese, the actual percentage of being obese is called precision, and when classified as not obese, the percentage that is not obese can be known through precision, and the percentage of actual obese people classified as not obese can also be known through recall. In addition, if not obese, the ratio that is classified as not obese is the specificity, and if the person is not obese, the ratio that is classified as obese can also be known through specificity. Accuracy is the percentage of data that are correctly classified, such as a person who is obese is classified as obese and a person who is not obese is classified as not obese. We evaluated the accuracy and AUC of the classifiers.

**Table 4:** Some metrics for the evaluation of a classifier based on the confusion matrix.

**/Metric**	**Equation**
Accuracy	$\frac{TP+TN}{TP+TN+FP+FN}$
Specificity	$\frac{TN}{FP+TN}$
Precision	$\frac{TP}{TP+FP}$
Recall (sensitivity)	$\frac{TP}{TP+FN}$
F1-score	$\frac{2\times Precision\times recall}{Precision+recall}$

### 4.3. Age-specific obesity classification

The KNHANES is a national program that is designed to assess the health and nutritional status of adults and children in Korea. Obesity is defined as BMI above 25 according to the Asia-Pacific adult guidelines (12). In our data, we combined obese (BMI of >25) and overweight (BMI of >23 and BMI of <25) into our definition of obese. After removing missing values, there were around 21,100 participants (male 10,000 and female 11,100). As can be seen from Figure 1, the proportion of overweight and obese people from 2016 to 2018 was 58%, and in 2019, the proportion of people with BMI of >23 was 57%. After we split the dataset into two parts by gender, we divided both the male and female participants into three age groups (19–39, 40–59, and 60–79), respectively. Therefore, our dataset consists of six groups composed according to gender and age group.

To predict obesity, it is very important to identify the risk factors of obesity. So, we identified metabolic features that contribute to high BMI or obesity. First, appropriate metabolic variables were selected based on previous research, from which we identify those that principally contribute to BMI in the KNHANES. And even though we did not find any association with studies suggesting that pulse rate causes obesity, being overweight or obese is positively associated with pulse pressure, independently of age and gender (43). Finally, we used the mean of the random forest value and the sharp value and selected the eight most significant factors for each of the six age-specific groups. The selected features were slightly different for each of the six population groups. The selected features for each group are given in Figure 4. The features are ALT (SGPT), glucose (glu), triglyceride (TG), white blood cell (WBC), glycated hemoglobin (HbA1c), creatinine (cre), systolic (diastolic) blood pressure (sbp/sdp), AST (SGOT), cholesterol (chol), hematocrit (Hct), platelet (Bplt), and uric acid (UA). The features ALT (SGPT), triglyceride (TG), HbA1c, and UA are selected for all groups (Figure 4). For all three male groups, ALT (SGPT) was the most important feature in both the sharp value and random Forest. For the female participants, triglyceride (TG) was the most important feature, except for the 60–79 age group. In the 60–79 age female group, HbA1c was the most important feature. The feature creatinine (crea) was only selected from the 40 to 59 and 60 to 79 age male groups. The feature Ast was selected for only 19–39 age male group and a Bplt feature was selected for only 40–59 age female group. Note that the SMOTE technique is used for oversampling due to class imbalance in the dataset. The number of obese and normal classes seems imbalanced. Supplementary Table S1 gives more detailed imbalance information of the six datasets. The SMOTE technique with oversampling was used, which resulted in two new datasets that are 50% obese and 50% not obese. Next, we employed six different machine learning algorithms to predict age- and gender-specific obesity for adults (19–79 years) in Korea. The six machine learning algorithms were performed using the top eight features as shown in Figure 4.

**Figure 5:**
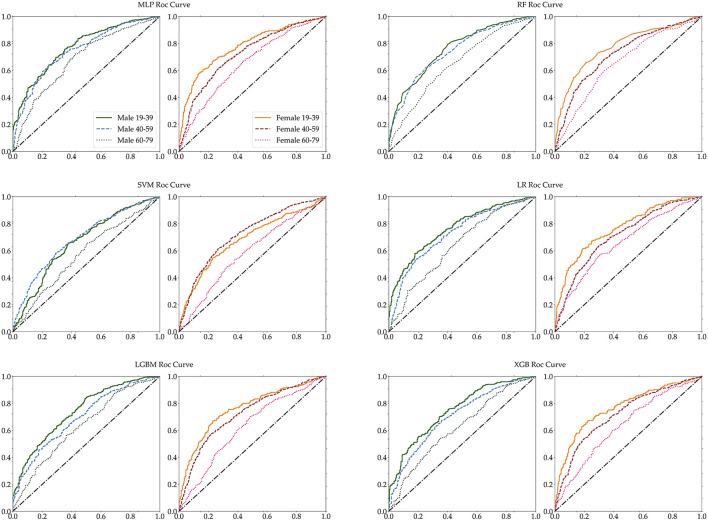
Age-specific ROC curves for each MLP, RF, SVM, LR, LGBM, and XGB. The left panel of each pair is male, while the right panel is female.

Furthermore, we measured the performance of these classifications in terms of several selected performance metrics. First of all, we have carried out 5-fold cross validation for train dataset as shown in Supplementary Table S2. Table 5 illustrates commonly used performance metrics by machine learning methods. Figure 5 shows the ROC curves of the performance of the algorithms for the three age groups per gender. The best performance classifier among the six groups is the MLP, which has the best performance for five age groups, all except for the 60–79 age female group. The MLP achieved an area under the ROC curve (AUC) of 0.78 in the 19–39 age female group, while achieving an AUC of 0.77 in the 19–39 age male group. However, in the 60–79 age group and both male and female groups, MLP achieved a lower AUC of 0.7. The RF and LF algorithms achieved the same AUC of 0.66 in the 60–79 age female group (Figure 5). In particular, MLP, RF, LGBM, and XGB achieved the same AUC of 0.72, while the LR achieved an AUC of 0.71 for the 40–59 age female group (Table 5). Those algorithms achieved better performance in each dataset. However, the SVM performed poorly (Table 5).

**Table 5:** Metric-wise performance of all classifiers used for each gender and age group.

**Algorithm**	**Evaluation**	**Male**			**Female**		
		**19–39**	**40–59**	**60–79**	**19–39**	**40–59**	**60–79**
MLP	Accuracy Recall Specificity Precision F1 score AUROC	0.70 0.72 0.68 0.81 0.76 0.77	0.67 0.63 0.76 0.86 0.73 0.75	0.60 0.56 0.67 0.75 0.64 0.68	0.73 0.68 0.76 0.59 0.63 0.78	0.67 0.66 0.69 0.69 0.67 0.72	0.62 0.64 0.58 0.74 0.69 0.65
RF	Accuracy Recall Specificity Precision F1 score AUROC	0.72 0.77 0.61 0.79 0.78 0.76	0.72 0.80 0.53 0.80 0.80 0.74	0.64 0.74 0.46 0.71 0.72 0.65	0.76 0.57 0.85 0.67 0.61 0.78	0.67 0.65 0.69 0.70 0.67 0.72	0.66 0.78 0.44 0.72 0.75 0.66
SVM	Accuracy Recall Specificity Precision F1 score AUCROC	0.65 0.70 0.53 0.74 0.72 0.66	0.66 0.70 0.55 0.79 0.74 0.69	0.60 0.69 0.44 0.68 0.68 0.58	0.69 0.53 0.78 0.55 0.54 0.68	0.67 0.63 0.70 0.70 0.66 0.72	0.58 0.62 0.51 0.70 0.66 0.59
LR	Accuracy Recall Specificity Precision F1 score AUROC	0.67 0.63 0.76 0.83 0.71 0.76	0.65 0.62 0.72 0.84 0.71 0.73	0.57 0.54 0.64 0.72 0.61 0.64	0.73 0.65 0.77 0.59 0.62 0.77	0.67 0.64 0.70 0.70 0.67 0.71	0.61 0.58 0.67 0.76 0.66 0.66
LGBM	Accuracy Recall Specificity Precision F1 score AUROC	0.70 0.80 0.53 0.76 0.78 0.74	0.71 0.86 0.38 0.76 0.81 0.69	0.62 0.73 0.41 0.69 0.71 0.63	0.74 0.53 0.84 0.64 0.58 0.76	0.67 0.65 0.69 0.69 0.67 0.72	0.65 0.79 0.39 0.70 0.75 0.62
XGB	Accuracy Recall Specificity Precision F1 score AUROC	0.69 0.77 0.55 0.76 0.77 0.75	0.71 0.84 0.40 0.76 0.8 0.70	0.61 0.72 0.40 0.68 0.7 0.62	0.75 0.58 0.83 0.64 0.61 0.77	0.66 0.65 0.67 0.68 0.67 0.72	0.64 0.78 0.39 0.7 0.74 0.64

The accuracy of each classifier differed for male and female participants, and the accuracy of female participants was higher than the accuracy of male participants for the 19–39 and 60–79 age groups. The RF algorithm achieved the best accuracy in all six groups, but for two groups of female and the male 60–79 age group, it accomplished below 0.7 accuracy. For male participants, RF achieved a higher 0.72 accuracy in the 40–59 age group and achieved 0.72 accuracy in the 19–39 age group. Across both genders, all algorithms achieved below 0.7 accuracy for the 60–79 age group. Table 5 shows that the SVM classifier achieved lower accuracy for all age groups and gender, and lost the performance of each of the six algorithms. The performance ability is determined according to its accuracy, sensitivity, specificity, precision, recall, F1-score, and ROC-AUC for the six age groups. The accuracy and AUC were the highest in the female aged 19–39 age group. The best algorithm for accuracy was RF, and for AUC the best was the MLP and LR algorithms.

**Figure 6:**
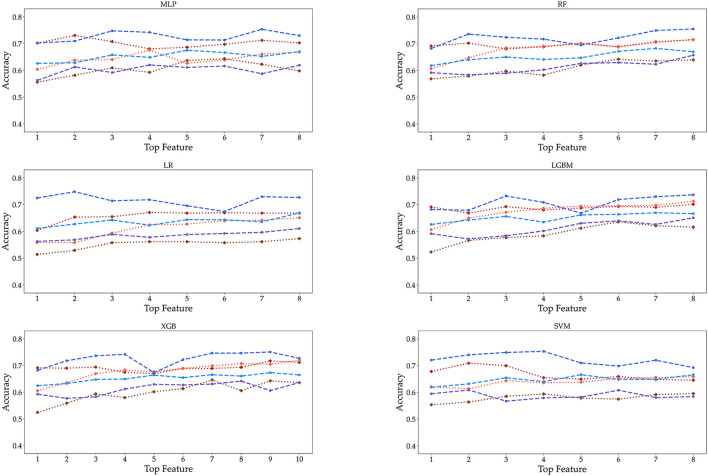
Accuracy is displayed as the number of features from 1 to 8 under three age groups and two genders. These features are extracted from the RF feature importance and mean absolute SHAP values in Figure 4.

Finally, as the features changed up to the top eight, the accuracy was measured by performing each algorithm for the six datasets (Figure 6). Even if the number of features increased to (5, 6, 7, up to 8), we cannot obtain the relationship between the accuracy and the number of features. Meanwhile, the accuracy of SVM tended to decrease in the female 19–39 age group. Also, the accuracy of algorithms shows little increase up to the top five or six. In the case of male participants, the 19–39 and 40–59 age groups tend to show a slight increase in the RF and LGBM algorithms. However, the algorithmic accuracy of the datasets did not change significantly depending on the number of the top eight or more features. We also carried out classification using KNN and GBM methods, and the results are given in Supplementary Figures S1, S2 and Supplementary Tables S3, S4.

## 5. Discussions

This study identifies risk factors for obesity in adults among the extensive dataset provided by the KDCA from 2016 to 2019 in Korea. First, in all six datasets, each of the top eight features was selected, which are shown in Figure 4. The eight features obtained by the mean of random forest value and mean absolute Shapley values include ALT (SGPT), glucose, triglyceride, white blood cell, glycated hemoglobin, creatinine, systolic (diastolic) blood pressure, AST (SGOT), cholesterol, hematocrit, platelet, and uric acid. For the three male datasets, the ALT (SGPT) was the most important feature in the mean of sharp value and random forest. For female datasets, triglyceride was the most important feature, except for the 60–79 age group. In the 60–79 age female group, HbA1c was the most important feature. The feature creatinine was only a top factor for the male 40–59 and 60–79 age groups.

Further research needs to be carried out using these factors with individual variances. Well-known factors for predicting obesity are age, gender, waist circumference, and race. These factors state the consequences of obesity. Moreover, various factors that cause obesity have been studied, and there have been many predictive studies using factors that affect obesity (1, 50, 55). Although a few researchers have included metabolic factors as key features in predicting obesity, most researchers have used individual factors, and individual lifestyle/behavioral and environmental factors as essential features (1, 5, 21). We identified essential metabolic factors affecting obesity and performed machine learning utilizing metabolic factors as key features. Our performance could not be compared with other research results, because the selected features are different (1, 56, 57).

In fact, there are other important factors that might influence obesity, such as food consumption, food production, physical activity, social psychology, genetic, or physiological and cultural influences, that were not included in our analysis because they are not suited for a machine learning approach. In addition, we used the KNHANES as data, but medical diagnostic records were not used.

Therefore, one of the main limitations of the study is that our features are not yet standard features for predicting obesity. It is important to evaluate and build predictive models for obesity using common risk factors. It would be desirable to improve the standardized of common factors affecting obesity because the current works have all dealt with different features. Another limitation of our model is the imbalanced data set from the KDCA, which contains a larger high-BMI population (people with BMI >23) than low-BMI population (people with BMI <23). A limitation of our study is that it did not use features such as gut microbiota composition, physiological, psychological, genetic, and environmental factors that cause obesity. Among the risk factors for obesity, we only used factors involved in metabolism as risk factors. We need to establish a set of risk factors for obesity in adults among the available study variables from those domains. Future research needs to identify and integrate risk factors in these areas. To facilitate future research, one needs to identify and integrate risk factors in these areas, and common features to predict obesity are required since there are many various factors that cause obesity. Hence, if standard factor assumptions are possible, common features to predict obesity may be used to carry out more elaborate predictions and sophisticated mathematical analysis.

## 6. Conclusion

We have investigated age- and gender-specific factors for obesity prediction. The top four significant features in all age-gender groups for predicting obesity are triglycerides, ALT (SGPT), glycated hemoglobin, and urine acid. For all three male groups, ALT (SGPT) was the most important feature in both the sharp value and random Forest. For the female groups, triglyceride (TG) was the most important feature, except for the 60–79 age group. In the 60–79 age female group, HbA1c was the most important feature. The feature Creatinine (crea) was only selected from the 40 to 59 and 60 to 79 age male groups while this creatinine was not important in all female and the 19–39 male group. Our findings show that the risk factors for obesity are sensitive to age and gender under different Machine Learning algorithms. The algorithms perform best for the male and female 19–39 age groups, with over 70% accuracy and AUC while for the 60–79 groups they show around 65% accuracy and AUC. Both the male and female 40–59 age groups achieved the highest performance of over 70% in AUC, but the females achieved lower than 70% in accuracy. For both classifiers and age groups, there is no big difference in accuracy when the number of features is more than six, except that the accuracy ratio decreased in the female 19–39 age group.

## Data availability statement

The datasets presented in this study can be found in online repositories. The names of the repository/repositories and accession number(s) can be found below: http://biomath.khu.ac.kr/index.php/resources/.

## Author contributions

JJ conducted the experiments. SL and CO wrote this manuscript and analyzed the results. All authors have read and agreed to the published version of the manuscript.

## Footnotes

^1^ https://knhanes.kdca.go.kr/knhanes
