# Supplementary material for: Age-specific risk factors for the prediction of obesity using a machine learning approach
Source: Front Public Health. 2023 Jan 17;10:998782. doi: 10.3389/fpubh.2022.998782 (PMC9887184; doi:10.3389/fpubh.2022.998782)
Supplement: Supplementary file 1 [file Data_Sheet_1.pdf]

Supplementary Material

SUPPLEMENTARY TABLES AND FIGURES

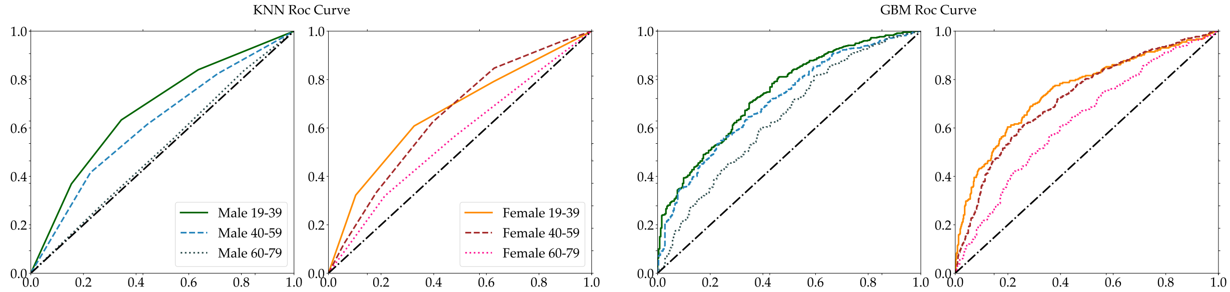

Figure S1: ROC curve with age-specific results are compared for both male and female.

Table S1. Number of male and female participants (normal/obesity).

No of features	Male			Female		
	19-39	40-59	60-79	19-39	40-59	60-80
Top 5	2712 (941/1771)	3851 (1069/2782)	3243 (1128/2115)	2681 (1756/925)	4656 (2282/2374)	4064 (1401/2663)
Top 6	2712 (941/1771)	3812 (1063/2749)	3225 (1126/2099)	2681 (1756/925)	4644 (2277/2367)	4063 (1401/2662)
Top 7	2706 (938/1768)	3812 (1063/2749)	3197 (1120/2077)	2677 (1755/922)	4644 (2277/2367)	4063 (1401/2662)
Top 8	2705 (938/1767)	3802 (1061/2741)	3197 (1120/2077)	2675 (1755/920)	4644 (2277/2367)	4063 (1401/2662)
Top 9	2705 (938/1767)	3802 (1061/2741)	3197 (1120/2077)	2675 (1755/920)	4644 (2277/2367)	4063 (1401/2662)
Top 10	2705 (938/1767)	3799 (1061/2738)	3191 (1118/2073)	2675 (1755/920)	4633 (2274/2359)	4054 (1401/2653)

Table S2. Cross validation score(mean accuracy, standard deviation) of algorithms.

Algorithm	Male			Female		
	19-39	40-59	60-80	19-39	40-59	60-80
MLP	0.72 $\pm$0.01	0.69 $\pm$0.02	0.64 $\pm$0.01	0.72 $\pm$0.02	0.69 $\pm$0.02	0.65 $\pm$0.02
RF	0.79 $\pm$0.04	0.82 $\pm$0.04	0.74 $\pm$0.05	0.80 $\pm$0.05	0.69 $\pm$0.02	0.76 $\pm$0.06
LR	0.71 $\pm$0.01	0.65 $\pm$0.02	0.62 $\pm$0.02	0.71 $\pm$0.02	0.67 $\pm$0.02	0.62 $\pm$0.03
LGBM	0.78 $\pm$0.07	0.81 $\pm$0.10	0.73 $\pm$0.06	0.80 $\pm$0.07	0.68 $\pm$0.03	0.73 $\pm$0.09
XGB	0.78 $\pm$0.06	0.80 $\pm$0.08	0.73 $\pm$0.06	0.78 $\pm$0.06	0.69 $\pm$0.02	0.74 $\pm$0.08
SVM	0.78 $\pm$0.05	0.77 $\pm$0.03	0.72 $\pm$0.05	0.74 $\pm$0.04	0.68 $\pm$0.02	0.71 $\pm$0.05
GBM	0.79 $\pm$0.06	0.81 $\pm$0.09	0.72 $\pm$0.06	0.80 $\pm$0.06	0.69 $\pm$0.02	0.74 $\pm$0.10
KNN	0.75 $\pm$0.04	0.76 $\pm$0.03	0.69 $\pm$0.04	0.76 $\pm$0.04	0.64 $\pm$0.03	0.70 $\pm$0.04

Table S3. Detailed specifications for the eight algorithms. The order of [,] is according to male age and female age.

Algorithms	Specification of algorithms
KNN	Number of neighbor = [3, 3, 3, 3, 5, 3]
	Power parameter, p = 2
	Metric : Minkowski
	Distances metric: Minkowski distance= $(\sum_{i=1}^{k}(\lvert x_i - y_i \rvert)^q)^{1/q}$
GBM	Maximum depth = [10, 10, 10, 10, 2, 10]
	num. estimators = [100, 100, 100, 100, 70, 100]

Table S4. Evaluations of GBM and KNN for 2 algorithms.

Algorithm	Evaluation	Male			Female		
		19-39	40-59	60-80	19-39	40-59	60-80
GBM	Accuracy	0.71	0.72	0.64	0.73	0.67	0.64
	Recall	0.79	0.86	0.77	0.54	0.64	0.79
	Specificity	0.56	0.39	0.42	0.83	0.69	0.36
	Precision	0.77	0.77	0.70	0.63	0.69	0.69
	F1 score	0.78	0.81	0.73	0.58	0.67	0.73
	AUROC	0.74	0.71	0.64	0.75	0.72	0.63
KNN	Accuracy	0.64	0.60	0.52	0.65	0.62	0.55
	Recall	0.63	0.62	0.56	0.61	0.63	0.56
	Specificity	0.66	0.56	0.46	0.67	0.60	0.52
	Precision	0.78	0.76	0.64	0.49	0.63	0.68
	F1 score	0.70	0.68	0.60	0.54	0.63	0.62
	AUCROC	0.67	0.62	0.51	0.67	0.65	0.56

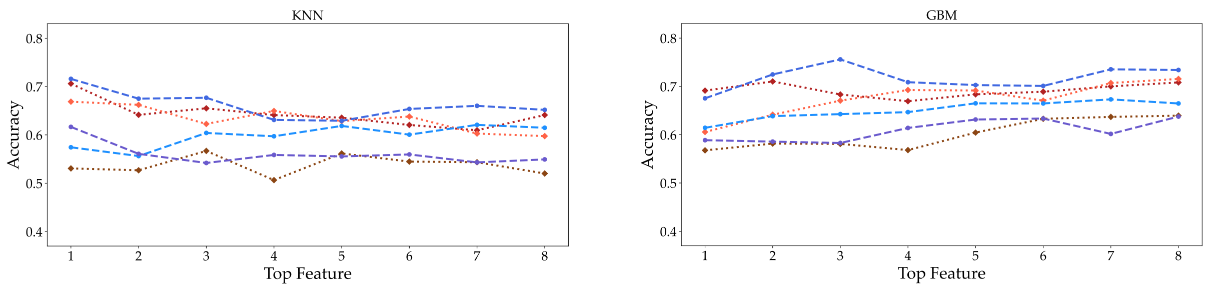

Figure S2: Accuracy is displayed as the number of features from 1 to 8 under three age groups and two genders.
